# Potential Common Genetic Risks of Sporadic Parkinson’s Disease and Amyotrophic Lateral Sclerosis in the Han Population of Mainland China

**DOI:** 10.3389/fnins.2021.753870

**Published:** 2021-10-11

**Authors:** Yi Lu, Wenzhi Chen, Caihui Wei, Yu Zhu, Renshi Xu

**Affiliations:** ^1^Department of Neurology, The First Affiliated Hospital of Nanchang University, Nanchang, China; ^2^Department of Neurology, Jiangxi Provincial People’s Hospital, Affiliated People’s Hospital of Nanchang University, Nanchang, China

**Keywords:** sporadic Parkinson’s disease, amyotrophic lateral sclerosis, common genetic risk, polymorphism, pathogenesis

## Abstract

Sporadic Parkinson’s disease (sPD) and sporadic amyotrophic lateral sclerosis (sALS) are neurodegenerative diseases characterized by progressive and selective neuron death, with some genetic similarities. In order to investigate the genetic risk factors common to both sPD and sALS, we carried out a screen of risk alleles for sALS and related loci in 530 sPD patients and 530 controls from the Han population of Mainland China (HPMC). We selected 27 single-nucleotide polymorphisms in 10 candidate genes associated with sALS, and we performed allelotyping and genotyping to determine their frequencies in the study population as well as bioinformatics analysis to assess their functional significance in these diseases. The minor alleles of rs17115303 in DAB adaptor protein 1 (*DAB1*) gene and rs6030462 in protein tyrosine phosphatase receptor type T (*PTPRT*) gene were correlated with increased risk of both sPD and sALS. Polymorphisms of rs17115303 and rs6030462 were associated with alterations in transcription factor binding sites, secondary structures, long non-coding RNA interactions, and nervous system regulatory networks; these changes involved biological processes associated with neural cell development, differentiation, neurogenesis, migration, axonogenesis, cell adhesion, and metabolism of phosphate-containing compounds. Thus, variants of *DAB1* gene (rs17115303) and *PTPRT* gene (rs6030462) are risk factors common to sPD and sALS in the HPMC. These findings provide insight into the molecular pathogenesis of both diseases and can serve as a basis for the development of targeted therapies.

## Introduction

Sporadic Parkinson’s disease (sPD) is a disorder caused by the progressive degeneration of dopaminergic (DA) neurons in the substantia nigra (SN) projecting to the striatum and containing cytoplasmic inclusions (Spillantini et al., 1997) that are mainly composed of α-synuclein protein (Stefanis, 2012). The major clinical features of sPD are tremor, rigidity, bradykinesia, and gait and postural abnormalities, as well as non-motor symptoms such as depression, anxiety, insomnia, and autonomic nervous system disorder resulting from damage to DA and non-DA neurons in the nigrostriatal pathway (Dauer and Przedborski, 2003). The observation that DA neuron terminals in the striatum is affected to a greater extent than SN DA neurons has led to the suggestion of a “retrograde” mechanism of neuron death in sPD (Dauer and Przedborski, 2003; Appel et al., 2010). However, the exact pathogenesis of sPD is not fully understood, although genetic factors are known to play a major role (Helley et al., 2017; Koros et al., 2017).

Amyotrophic lateral sclerosis (ALS) is a motor neuron disease with an adult-onset and progressive course that involves the degeneration of upper and lower motor neurons in the brain, brainstem, and spinal cord, leading to limb and even whole body muscle atrophy with different degrees of paralysis, dysarthria, and/or dysphagia. Sporadic (s)ALS patients often die from respiratory failure caused by respiratory muscle paralysis 3–5 years after disease diagnosis (Haverkamp et al., 1995; Boillée et al., 2006), and the median interval from diagnosis to death was 29 months (Haverkamp et al., 1995). Although the precise etiology of sALS is not known, genetic and environmental factors are thought to contribute (Alfahad and Nath, 2013; Corcia et al., 2017; Sher, 2017; Yu and Pamphlett, 2017). Denervation at the neuromuscular junction is observed prior to the loss of motor neurons in the spinal cord, suggesting that the pathogenesis of sALS originates at the distal axon and proceeds in a retrograde manner similar to what is observed in sPD (Dauer and Przedborski, 2003; Fischer et al., 2004). As in sPD, neuronal inclusions are a prominent pathologic feature of sALS and involve the deposition of abnormal proteins in neurons of the cerebrum, brainstem, and spinal cord (Blokhuis et al., 2013; Takeda, 2018); additionally, damage to other neural cell types is observed in both diseases (Li et al., 2016; Lu et al., 2016; Liang et al., 2017; Zhang et al., 2018). sALS affects motor neurons and glia in brain regions outside the cerebral cortex including the bulbus medullae and anterior horn of the spinal cord (Cozzolino et al., 2008). Given these observations, it is possible that sPD and sALS share common pathogenic mechanisms and genes (Gomes-Trolin et al., 2002; Hu et al., 2017; Chen et al., 2018a, b; Yuan et al., 2018).

The majority of PD and ALS cases (90–95%) are sporadic. Insight into the pathogenesis of sPD and sALS has been gained from animal models of genetic and toxin-induced forms of these diseases. Mutations in the gene encoding Cu^2+^/Zn^2+^ superoxide dismutase 1 (SOD1) are the most common cause of familial ALS, which has been studied using a mouse (m)SOD1 transgenic animal model (Rosen et al., 1993; Boillée et al., 2006). Neurotoxins that induce the signs and symptoms of sPD (Langston et al., 1999) have also been used to establish models to investigate the molecular basis of DA cell damage. Findings from preclinical and clinical studies indicate that mitochondrial dysfunction, increased production of reactive oxygen species, protein misfolding and aggregation, and dysregulation of the ubiquitin proteosome pathway cause neurodegeneration in both sPD and sALS (Dauer and Przedborski, 2003; Boillée et al., 2006). However, a detailed understanding of the precipitating molecular events is lacking. Experiments in mSOD1 mice have suggested that the observed neuronal death is a non-cell autonomous process involving microglia, astrocytes, and T cells (Clement et al., 2003).

**FIGURE 1:**
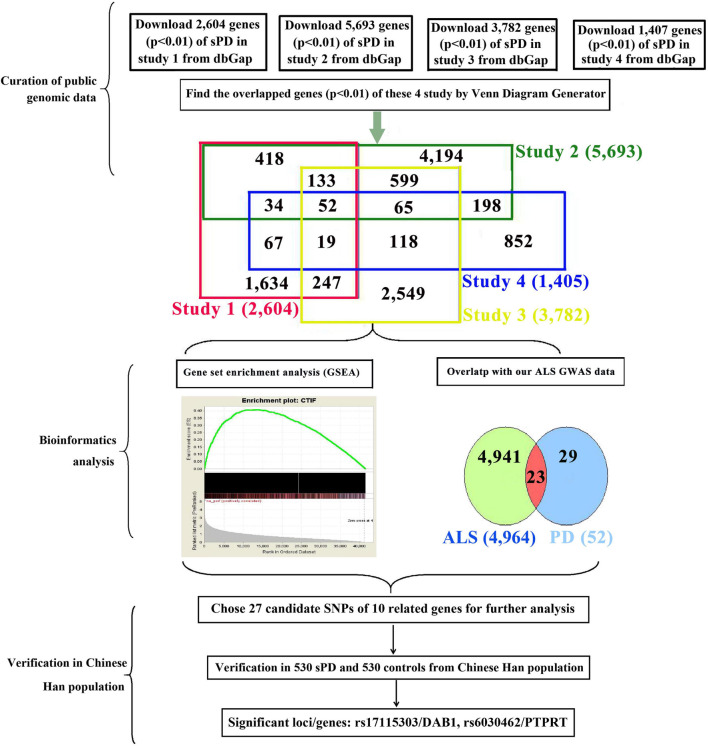
Study design and procedures. To identify common pathogenic genes between sPD and sALS, we downloaded four independent GWAS datasets from the dbGap database of the NCBI, mapped all SNPs to genic regions, and then preformed an integrative analysis to scan for common genetic factors related to sPD. We also compared the 52 candidate genes with our previous sALS data from a GWAS of the HPMC and found 23 that were significant in both sPD and sALS. We selected 27 loci in 10 related genes for validation in an independent cohort of 530 sPD patients and 530 neurologically normal control subjects at the Affiliated Hospital of Nanchang University. *DAB1* rs17115303 and *PTPRT* rs6030462 were identified as being closely associated with both sPD and sALS. sPD, sporadic Parkinson’s disease; sALS, sporadic amyotrophic lateral sclerosis; GWAS, genome-wide association study; NCBI, National Center for Biotechnology Information; SNPs, single-nucleotide polymorphisms; HPMC, Han population of mainland China.

The current known genes associated with the sPD and sALS pathogenesis included approximately more than 5,000; the most potential common genes were 23 genes according to our bioinformatics analysis with our previous genome-wide association study (GWAS) analysis (Figure 1). Therefore, we speculated that common genetic mechanisms underlie the pathogenesis of both sPD and sALS. To test this hypothesis, we screened genes known to be associated with sALS and related loci and selected 27 single-nucleotide polymorphisms (SNPs) in 10 candidate genes for analysis. We investigated whether these SNPs were present in sPD patients from the Han population of Mainland China (HPMC).

## Materials and Methods

### Participants

sPD patients and control subjects (*n* = 530 each) from the HPMC were recruited at the First Affiliated Hospital of Nanchang University. A modified version of the United Kingdom Parkinson’s Disease Society Brain Bank Clinical Diagnostic Criteria (Gibb and Lees, 1988; Albanese, 2003) was used for sPD diagnosis. The patients exhibited at least two of following signs: resting tremor, rigidity, bradykinesia, and gait or postural disorder. All participants underwent the same clinical and laboratory examinations. We also reviewed general medical history, PD family history, related diseases in first-degree relatives, and potential exposure to toxicants related to sPD; and we performed magnetic resonance imaging of the brain and spinal cord to exclude other neurologic diseases with clinical manifestations similar to those of sPD such as tumors, demyelinating disorders, hydrocephalus, and cervical myelopathy. All procedures were approved by the institutional review board of the human ethics committee of the First Affiliated Hospital of Nanchang University. Written informed consent was obtained from all participants or their legal guardians before patient enrollment. All experimental methods were in compliance with the principles of the Declaration of Helsinki.

### Integrative Analysis of Genetic Data From Genome-Wide Association Study Datasets

We downloaded the genome-wide *p*-value data for sPD-related GWASs from the dbGap database of National Center for Biotechnology Information (NCBI).^1^ The GWAS data were from four studies of American and Caucasian (Germany and the United Kingdom) populations (Maraganore et al., 2005; Fung et al., 2006; Simón-Sánchez et al., 2009; Pankratz et al., 2011) involving 7,025 sPD cases (including 816 familial PD cases) and 10,631 controls (Figure 1). We performed pairwise comparisons of the gene sets of the four studies based on the *p*-values. A total of 2,779 SNPs in 52 genes were identified (Supplementary Table 1). We compared the 52 genes with our previous sALS data from a GWAS of the HPMC (Xie et al., 2014) and found 23 that were common to both sPD and sALS. We selected 27 SNPs in 10 candidate genes for allelotyping and genotyping (Supplementary Tables 2–4).

### Allelotyping and Genotyping of Sporadic Parkinson’s Disease Case–Control Samples

DNA was isolated from white blood cells of sPD patients and control subjects using a DNA extraction kit (Fastgene Co., Shanghai, China) according to the manufacturer’s instructions. The samples were verified by electrophoresis on a 1.5% agarose gel to ensure that there was no RNA contamination or DNA degradation. Samples of intact genomic DNA were selected for allelotyping and genotyping analyses. For the former, the false-positive report probability along with the relative allele frequency was calculated for loci surrounding candidate genes to estimate the confidence interval (CI) and *p*-value corresponding to the odds ratio (OR) score (Wacholder et al., 2004). Candidate polymorphic loci were genotyped using the Sequenom Mass ARRAY iPLEX platform (Agena Bioscience, San Diego, CA, United States). All experimental procedures including primer design and synthesis and genotyping were carried out by I-conoene Biotechnology Co. (Wuhan, China). The methods of genotyping were performed by Sequenom MassArray. For quality control, 5% of the DNA samples were randomly selected; and the assay was repeated, with a concordance rate of 100% (Su et al., 2015).

### Statistical Analysis

Demographic data (continuous variables) of sPD patients and control subjects are expressed as mean ± standard deviation (SD). Differences between sPD patients and control subjects were evaluated with the Student’s *t*-test, χ^2^ test, or Fisher’s exact test using SPSS v17.0 (SPSS Inc., Chicago, IL, United States). The allele frequency of loci was estimated using the ratio of signal intensities of each allele. Differences in allele frequencies between sPD patients and controls were evaluated with the combined *Z* test. Statistical analyses were performed with SAS v9.1.3 (SAS Institute, Cary, NC, United States). A *p*-value < 0.05 was considered statistically significant.

### Functional Prediction of Candidate Single-Nucleotide Polymorphisms in Both Sporadic Parkinson’s Disease and Sporadic Amyotrophic Lateral Sclerosis

#### Prediction of Transcription Factor Binding Sites

The sequences from 100 bp upstream to 100 bp downstream of DAB adaptor protein 1 (*DAB1*) rs17115303 and protein tyrosine phosphatase receptor type T (*PTPRT*) rs6030462 were analyzed with NHRscan software (Sandelin and Wasserman, 2005). Transcription factor binding sites in the sequences 1,500 bp upstream of *DAB1* rs17115303 and *PTPRT* rs6030462 were predicted using Mscan software (Alkema et al., 2004).

#### Prediction of Secondary Structure

The secondary structure of the sequences 100 bp upstream to 100 bp downstream of *DAB1* rs17115303 and *PTPRT* rs6030462 was analyzed using Mfold software (Zuker, 2003).

### Binding Potential of Long Non-coding RNAs

Human long non-coding RNA (lncRNA) sequences in the GRCh38 assembly were downloaded from the Ensembl Genome Browser website^2^ and formatted as a database. Intron sequences from 50 bp upstream to 50 bp downstream of *DAB1* rs17115303 and *PTPRT* rs6030462 were extracted from GRCh38 and searched in the database by BLAST with an *e*-value cutoff of 1,000 to obtain high-scoring segment pair (HSP) hits with the complementary intron sequence.

### Regulatory Network Construction

A nervous system regulatory network was constructed using transcription factors that bind to the promoter region of *DAB1* [V-rel avian reticuloendotheliosis viral oncogene homolog A (*RELA*), myoblast determination protein 1 (*MYOD1*), and MDS1 and EVI1 complex locus (*MECOM*)] and *PTPRT* [Kruppel-like factor 4 (*KLF4*) and Ras-responsive element binding protein 1 (*RREB1*)] genes along with risk genes for ALS [fusion, derived from T(12;16) malignant liposarcoma (*FUS*), uncoordinated 13 homolog A (*UNC13A*), activator protein complex subunit beta (*APB*), peroxisome proliferator-activated receptor gamma coactivator 1-alpha (*PPARGC1A*), hemoprotein 2B (*HMP2B*), peripherin (*PRPH*), matrin 3 (*MATR3*), triggering receptor expressed on myeloid cells 2 (*TREM2*), coiled-coil-helix-coiled-coil-helix domain-containing 10 (*CHCHD10*), dynactin subunit 1 (*DCTN1*), optineurin (*OPTN*), TANK-binding kinase 1 (*TBK1*), ubiquilin 2 (*UBQLN2*), D-amino acid oxidase (*DAO*), neurofilament heavy (*NEFH*), *FIG4*, retinoblastoma-binding 4 (*RBB4*), *SOD1*, *HNRNPA1*, valosin-containing protein (*VCP*), chromosome 9 open reading frame 72 (*C9ORF72*), TAR DNA-binding protein (*TARDBP*), sequestosome 1 (*SQSTM1*), angiogenin (*ANG*), paraoxonase 1 (*PON1*), *PON*2, *PON3*, profiling 1 (*PFN1*), and ataxin 2 (*ATXN2*)] obtained from the OMIM database (OMIM 105400^3^).

### Gene Ontology Analysis of Candidate Genes

All 10 identified risk genes common to sPD and sALS in our study are listed in the Gene Ontology (GO) database.^4^ Functional prediction analyses were performed by Gene For Health Biotechnology Co. (Shanghai, China). We selected “hsapiens” as the organism and “hsapiens_gene_symbol” as the gene ID type when uploading the genes of interest and reference gene set. We selected “hsapiens_genome” as the reference set, *p* < 0.01 as the significance level, and hypergeometric as the statistical method.

## Results

### Demographic Characteristics of Participates

Studied populations were composed of a total of 530 sPD and 530 controls. The average age at the onset (sPD) was 58.72 ± 0.91. The average age at the enrollment (Controls) was 59.56 ± 0.92. The percent of males was 51.70%; that of control was 60%. The median (range) age of sPD was 60 (32–83) years, and that of control was 60 (31–83) years (Supplementary Table 5).

### Candidate Genes in Both Sporadic Parkinson’s Disease and Sporadic Amyotrophic Lateral Sclerosis

**FIGURE 2:**
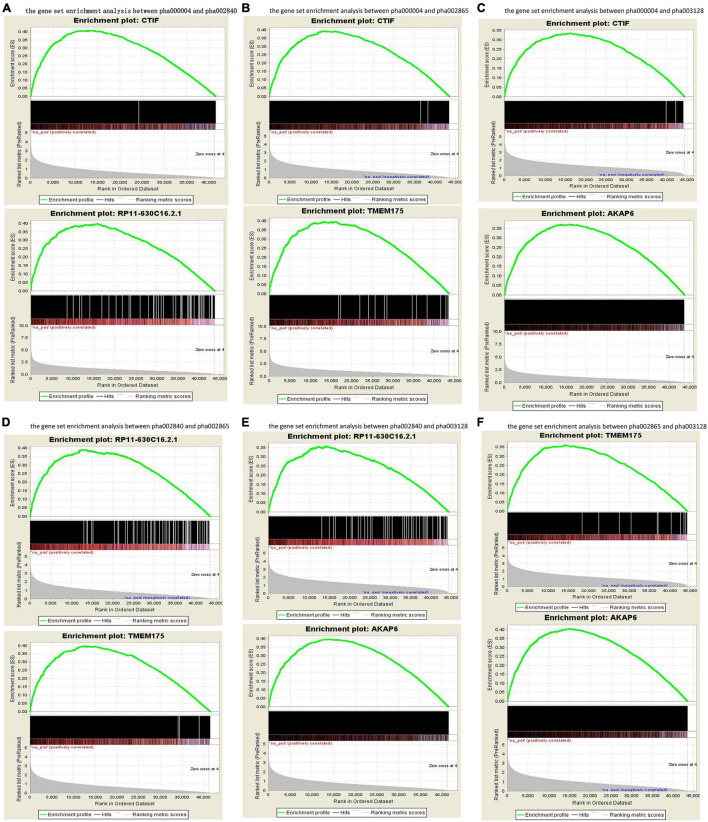
Gene set enrichment analysis of the four GWAS datasets. Pairwise comparisons of gene sets from four studies were performed based on *p*-values. **(A–C)** pha000004 was enriched compared with pha002840 **(A)**, pha002865 **(B)**, and pha003128 **(C)**. **(D,E)** pha002840 was enriched compared with pha002865 **(D)** and pha003128 **(E)**. **(F)** pha002865 was enriched compared with pha003128. These results confirm the reproducibility of the four studies and indicate that similar molecular mechanisms of sPD-related genes were found in all of the studies. GWAS, genome-wide association study; sPD, sporadic Parkinson’s disease.

**FIGURE 3:**
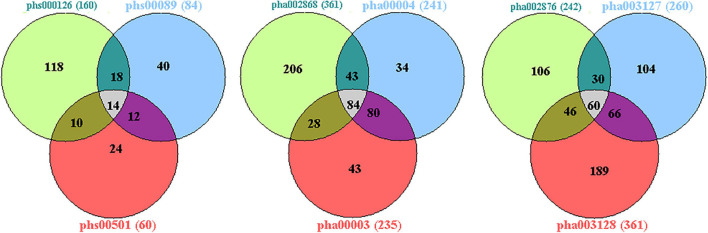
Venn diagram analysis of the four GWAS datasets. There were 43,474 candidate genes with at least one genotyped locus in study 1; 44,304 candidate genes in study 2; 43,699 candidate genes in study 3; and 41,458 candidate genes in study 4. There were 2,779 overlapping candidate genes between the four datasets by Venn diagram analysis; based on a nominal threshold *p*-value < 0.01, we identified 52 genes that were significantly associated with sPD. GWAS, genome-wide association study; sPD, sporadic Parkinson’s disease.

The published genetic data of GWAS dataset from four origins of non-HPMC composed of the United States, Germany, and the United Kingdom were from performed enrichment analysis, which showed that pha000004 was drastically enriched at the top of list from pha002840 [enrichment score (ES) = 0.409, normalized ES (NES) = 1.455, nominal (NOM) *p*-value < 10^–3^]. The significant enrichment of pha002840 in the pha000004 data further indicated the greater consistency across two genetic datasets (ES = 0.398, NES = 1.602, NOM *p*-value < 10^–3^) (Figure 2A); pha000004 was drastically enriched at the top of list from pha002865 (ES = 0.393, NES = 1.555, NOM *p*-value < 10^–3^). The significant enrichment of pha002865 in the pha000004 data further indicated the greater consistency across two genetic datasets (ES = 0.397, NES = 1.610, NOM *p*-value < 10^–3^) (Figure 2B); pha000004 was drastically enriched at the top of list from pha003128 (ES = 0.335, NES = 1.739, NOM *p*-value < 10^–3^). The significant enrichment of pha003128 in pha000004 data further indicated the greater consistency across two genetic datasets (ES = 0.372, NES = 1.520, NOM *p*-value < 10^–3^) (Figure 2C); pha002840 was drastically enriched at the top of list from pha002865 (ES = 0.390, NES = 1.521 NOM *p*-value < 10^–3^). The significant enrichment of pha002865 in the pha002840 data further indicated the greater consistency across two genetic datasets (ES = 0.398, NES = 1.410, NOM *p*-value < 10^–3^) (Figure 2D); pha002840 is drastically enriched at the top of list from pha003128 (ES = 0.357, NES = 1.825, NOM *p*-value < 10^–3^). The significant enrichment of pha003128 in pha2840 data further indicated the greater consistency across two genetic datasets (ES = 0.397, NES = 1.415, NOM *p*-value < 10^–3^) (Figure 2E). pha002865 was drastically enriched at the top of list from pha003128 (ES = 0.361, NES = 1.862, NOM *p*-value < 10^–3^). The significant enrichment of pha003128 in the pha002865 data further indicated the greater consistency across two genetic datasets (ES = 0.405, NES = 1.608, NOM *p*-value < 10^–3^) (Figure 2F). We obtained 2,779 SNPs of 52 genes from the enrichment results (Supplementary Table 1; Figure 3). Then we conducted an integrative analysis with our previously reported candidate genes of sALS from our GWAS data (Xie et al., 2014). Of these, our results showed that 23 genes were overlapped genes in both sPD and sALS; they might be the potential common pathogenic genes of both sPD and sALS.

### Verification in Han Population of Mainland China

**FIGURE 4:**
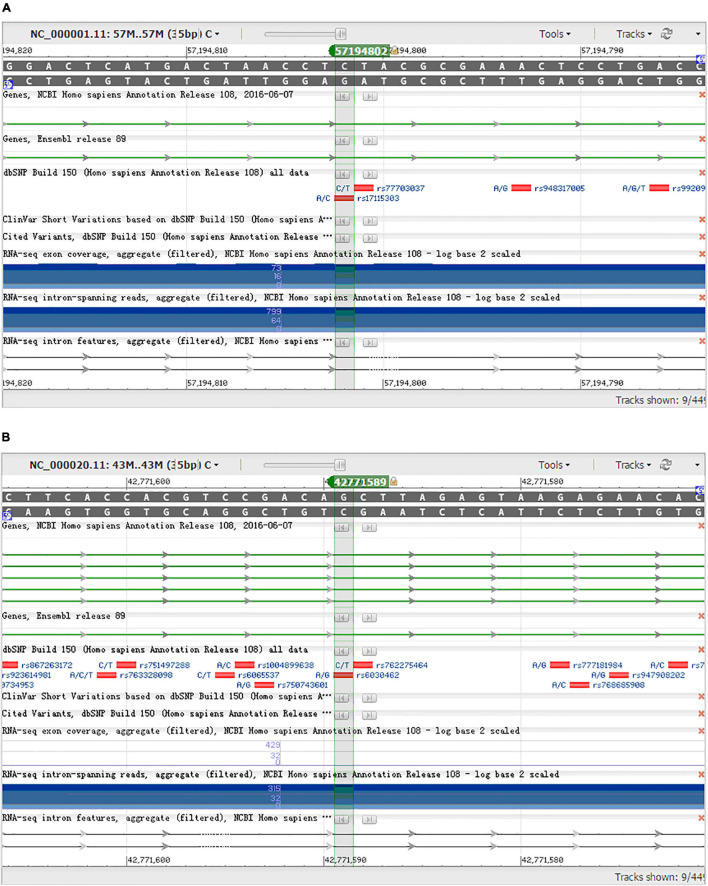
NCBI gene sequence positions of *DAB1* rs17115303 and *PTPRT* rs6030462. **(A)** *DAB1* gene located at chr1:57194802 with a C-to-A transversion on the reverse strand. **(B)** *PTPRT* gene located at chr20:42771589 with a G-to-A transition on the reverse strand. NCBI, National Center for Biotechnology Information.

From 23 genes of overlapped genes in both sPD and sALS, we chose 27 SNPs in 10 candidate genes for further allelotyping and genotyping analyses (Supplementary Tables 2–4). The result determined that two novel SNPs, DAB1 rs17115303 and PTPRT rs6030462, shared in both sPD and sALS. They both were SNPs of introns. Gene DAB1 base sequence is located in the chr1:57194802 position; DAB1 rs17115303 changed from C to A at the reverse strand (Figure 4A). Gene PTPRT base sequence is located in the chr20:42771589 position; PTPRT rs6030462 changed from G to A at the reverse strand (Figure 4B).

### Allelic and Genotypic Distribution of Two Novel Single-Nucleotide Polymorphisms in Cases and Controls and Analysis of Minor Allele Frequencies

Allelic and genotypic frequencies are summarized in Tables 1, 2. Two novel SNPs were identified, as follows: rs17115303 in *DAB1* gene and rs6030462 in *PTPRT* gene. The genotype frequency of rs17115303 differed significantly (*p* = 0.047) between patients and controls. The minor allele frequency (MAF) of rs17115303 was higher in the cases (8.8%) than in the controls (6.8%), and the difference was significant (*p* = 0.039). The minor A allele of rs17115303 (OR 1.42, 95% CI 1.024–1.970) exhibited an increased risk for the disease. C allele of rs17115303 (OR 0.704, 95% CI 0.508–0.976) exhibited a decreased risk for the disease. *p*-Value of rs6030462 genotype frequency was significant (*p* = 0.019) between patients and controls. The MAF of rs6030462 was higher in cases (44.2%) than in controls (41.3%), and the difference was not significant (*p* = 0.306). The minor A allele of rs6030462 (OR 1.125, 95% CI 0.899–1.407) exhibited an increased risk for the disease. G allele of rs6030462 (OR 0.889, 95% CI 0.711–1.112) exhibited a decreased risk for the disease. Minor alleles of rs17115303 in *DAB1* gene and rs6030462 in *PTPRT* gene were correlated factors with the disease, and they significantly increased the risk of disease.

**TABLE 1:** Two novel SNPs shown the nominal significance at *P* < 0.05 in this study.

SNP	Group	Genotypes			Genotype		MAF	Allelic *P* value	Allelic OR (95%CI)	Global MAF	Hap-Map CHB MAF
		
					χ^2^	*P* value					
DAB1		AA	CC	CA			A Allele				
rs17115303	Cases (*n* = 525)	3	436	86	6.097	0.047	0.088	0.039	A Allele:1.420 (1.024–1.970)	*A* = 0.0186	*A* = 0.0728
	Controls (*n* = 529)	0	462	67			0.068		C Allele:0.704 (0.508–0.976)		
PTPRT		AA	GG	GA			A Allele				
rs6030462	Cases (*n* = 513)	100	159	254	7.95	0.019	0.442	0.306	A Allele:1.125 (0.899–1.407)	*A* = 0.4872	*A* = 0.5825
	Controls (*n* = 226)	50	89	87			0.413		G Allele:0.889 (0.711–1.112)		

*Abbreviations: SNP = Single nucleotide polymorphism; MAF = Minor allele frequency; OR = Odds ratio; CI = Confidence interval; Global MAF: The data of 1000 genomes browser; CHB = Han Chinese in Beijing.*

*P value significance < 0.05.*

**TABLE 2:** Verified loci in Chinese Han populations of mainland.

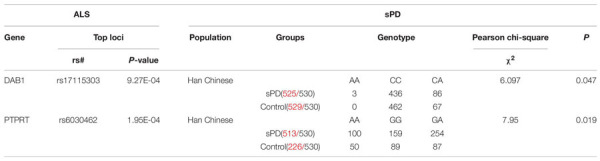

*Red values mean statistical number in 530 patients.*

### Biological Informational Analysis of DAB Adaptor Protein 1 rs17115303

**FIGURE 5:**
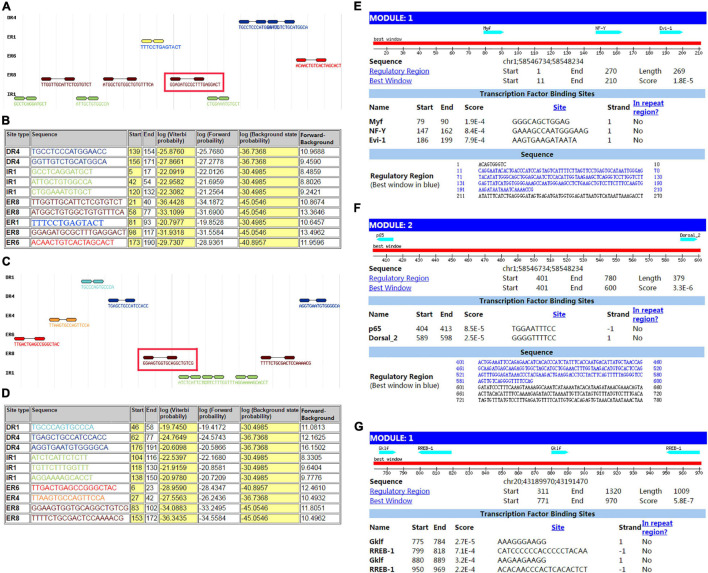
Predicted transcription factor binding sites in *DAB1* rs17115303 and *PTPRT* rs6030462. The sequences from 100 bp upstream to 100 bp downstream of *DAB1* rs17115303 and *PTPRT* rs6030462 were analyzed using NHRscan software. **(A)** The sequence from 98 to 117 of *DAB1* rs17115303 was predicted as the binding site fragment (ER8: GGAGATGCGCTTTGAGGACT; red box) with a mutation at base position 4. **(C)** The sequence from 83 to 102 of *PTPRT* rs6030462 was predicted as the binding site fragment (ER8: GGAAGTGGTGCAGGCTGTCG; red box), with a mutation at base position 19. **(B,D)** Statistical analysis of predicted binding sites of *DAB1* rs17115303 **(B)** and *PTPRT* rs6030462 **(D)**. **(E–G)** Predicted transcription factor binding sites of *DAB1* rs17115303 and *PTPRT* rs6030462 using NRHscan and Mscan software; transcription factor binding sites were predicted at 1,300–1,420 and 900–1,100 bp in the *DAB1* rs17115303 binding site fragment **(E,F)** and at 725–531 bp in the *PTPRT* rs6030462 binding site fragment. The mutations in *DAB1* rs17115303 and *PTPRT* rs6030462 were predicted to prevent transcription factor binding.

The sequence from upperstream 100 bp to downstream 100 bp of DAB1 rs17115303 mutation position was analyzed using NHRscan software. The position from 98 to 117 of sequence in the fragment of red frame in Figure 5A, ER8: GGAGATGCGCTTTGAGGACT, was predicted to be the binding site fragment, and the mutation site was at the fourth base position of binding fragment (Figures 5A,B). The sequence in the upstream 1,500 bp of DAB1 rs17115303 was applied to predicted transcription factor binding sites using the Mscan software. The binding site was predicted at distances from 1,300 to 1,420 bp and 900 to 1,100 bp upstream of the binding site; the predicted results based on the Mscan software showed that the DAB1 rs17115303 mutation position might be in the binding block (Figures 5E,F).

**FIGURE 6:**
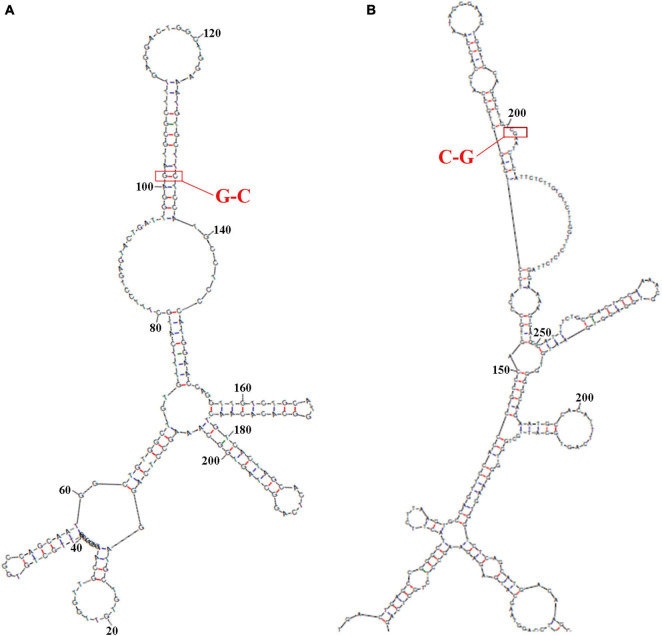
Predicted secondary structures of *DAB1* rs17115303 and *PTPRT* rs6030462. The secondary structure of the sequence from 100 bp upstream to 100 bp downstream of *DAB1* rs17115303 and *PTPRT* rs6030462 mutation positions were analyzed using Mfold software. **(A,B)** Secondary structure prediction for the introns of *DAB1* rs17115303 **(A)** and *PTPRT* rs6030462 **(B)**. The binding regions formed a hairpin structure. By overlaying observed SNPs (red triangles) and predicted binding sites (red lines) onto the secondary structure of *DAB1* rs17115303 and *PTPRT* rs6030462, it was determined that the hairpin structure may be required for protein binding. The SNPs of both genes were located in the cap region and were predicted to destabilize the hairpin, thereby affecting splicing. SNPs, single-nucleotide polymorphisms.

The secondary structure of sequence from the upperstream 100 bp to the downstream 100 bp of DAB1 rs17115303 mutation position was predicted using the Mfold software and visualized potential binding sites. The intron sequence from the upperstream 100 bp to the downstream 100 bp of DAB1 rs17115303 mutation position was extracted from the GRCh38 of human genome version. The result showed that the binding region formed a hairpin-like structure in the secondary structure. By overlaying observed SNPs and predicted binding sites on the secondary structure of intron, it was possible that the intron used a hairpin structure for the protein binding. The observed intronic SNP was located in the cap region, possibly harmed the stability of hairpin, and in turn influenced the splicing event (Figure 6A).

**FIGURE 7:**
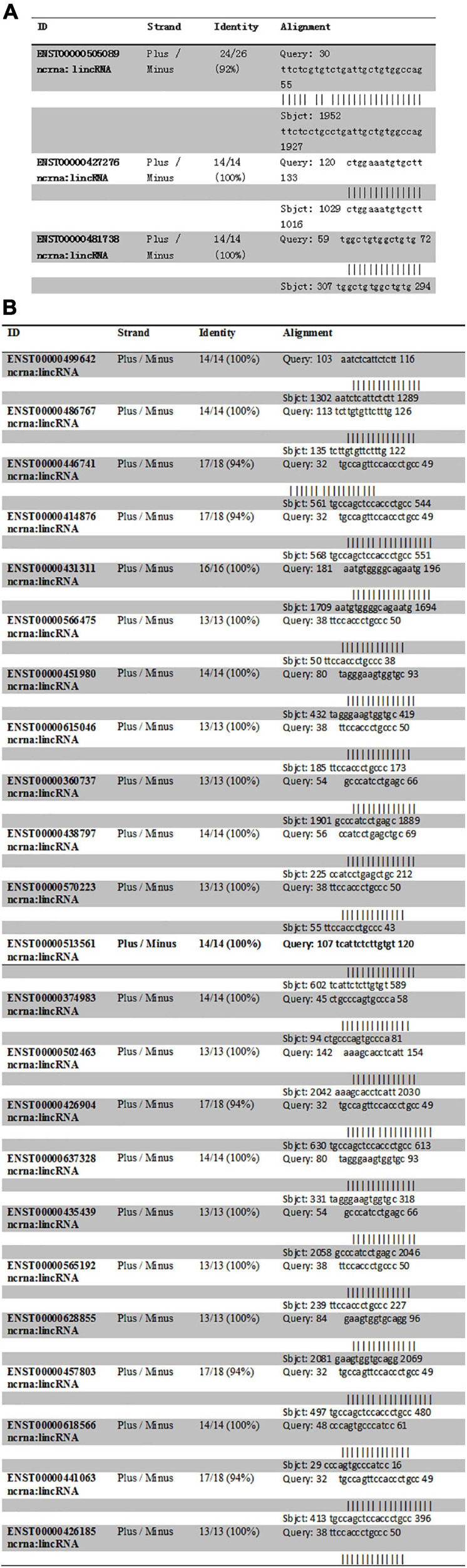
Predicted lncRNA binding sites in *DAB1* rs17115303 and *PTPRT* rs6030462. **(A,B)** Three lncRNAs were predicted to bind *DAB1* rs17115303 **(A)**, and 23 lncRNAs were predicted to bind *PTPRT* rs6030462 **(B)**. In both cases, the lncRNAs were presumed to bind the transcriptional intron and not the DNA strand. lncRNA, long non-coding RNA.

The intron sequence from the upperstream 50 bp to the downstream 50 bp of DAB1 rs17115303 mutation position was extracted from the version GRCh38 of human genome. The each sequence of lncRNA was applied to blast against the database. Blastn with *e*-value cutoff 1,000 was performed to obtain short HSP hits with the complemented intron sequence. The result obtained three lncRNA hits. Those hits with the opposite orientation compared with intron sequences (Plus/Minus) were filtered as the lncRNA that could bind the transcriptional intron, but not directed on the DNA strand (Figure 7A).

**FIGURE 8:**
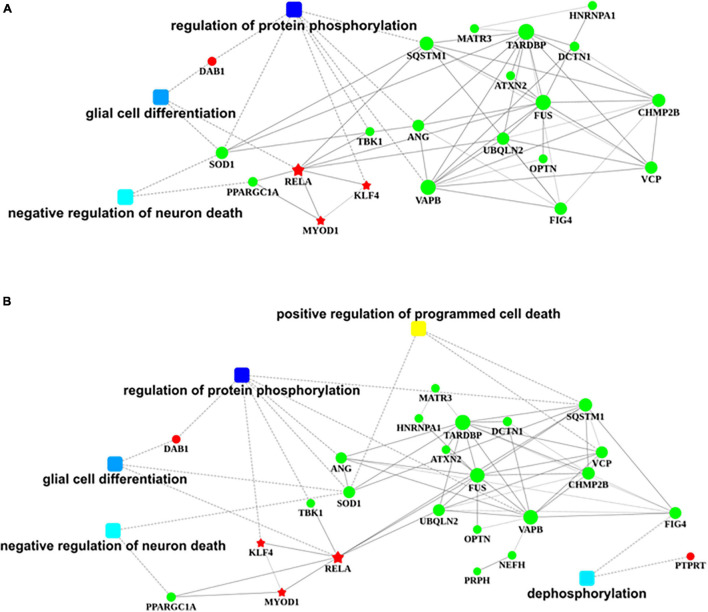
Regulatory networks of *DAB1* and *PTPRT*. **(A,B)** Nervous system regulatory networks of transcription factors binding to the promoter region of *DAB1* gene **(A)** and *PTPRT* gene **(B)** and genes related to ALS (OMIM 105400). The red pentagon represents transcription factors; the green node represents predisposing genes; squares represent biological processes; and lines indicate the relationships between transcription factors, genes, and biological processes. ALS, amyotrophic lateral sclerosis.

The nervous system regulation network building from transcription factors that bind to the promotor region of *DAB1* gene showed that *DAB1* gene mainly participated in the regulation of protein phosphorylation, the glial cell differentiation, and the regulation of neuron death, which are majorly involved in *SOD1*, *PPARGC1A*, *TBK1*, *VAPB*, *ANG*, and *SQSTM1* genes; their related genes including *MATR3*, *ATXN2*, *UBQLN2*, *TARDBP*, *FUS*, *OPTN*, *FIG4*, *HNRNPA1*, *DCTN1*, *CHMP2B*, and *VCP* genes; and KLF4, RELA, and MYOD1 transcription factors (Figure 8A).

### Biological Informational Analysis of Protein Tyrosine Phosphatase Receptor Type T rs6030462

The sequence from the upperstream 100 bp to the downstream 100 bp of PTPRT rs6030462 mutation position was analyzed using the NHRscan software. The position from 83 to 102 of sequence in ER8: GGAAGTGGTGCAGGCTGTCG was predicted to be the binding site fragment, and the mutation site was at the 19th base position of binding fragment (Figures 5C,D). The sequence in the upstream 1,500 bp of PTPRT rs6030462 was applied to transcription factor binding sites prediction using the Mscan software. The binding site was predicted at distances from 725 to 531 bp upstream of binding site; predicted results based on the Mscan software showed that the PTPRT rs6030462 mutation position might be in the binding block (Figure 5G). The secondary structure of sequence from upperstream 100 bp to downstream 100 bp of PTPRT rs6030462 mutation position was predicted using the Mfold software and visualized potential binding sites. The intron sequence from the upperstream 100 bp to the downstream 100 bp of PTPRT rs6030462 mutation position was extracted from the version GRCh38 of human genome. The result showed that the binding region formed a hairpin-like structure in the secondary structure. By overlaying observed SNPs and predicted binding sites on the secondary structure of the intron, it was possible that the intron used a hairpin structure for the protein binding. Observed intronic SNPs were located in the cap region, which possibly harmed the stability of hairpin and in turn influenced the splicing event (Figure 6B).

Human lncRNA sequences were downloaded from the version GRCh38 of Ensemble website and formatted as the database. The intron sequence from the upperstream 50 bp to the downstream 50 bp of PTPRT rs6030462 mutation position was extracted from the version GRCh38 of human genome. Each sequence of lncRNA was applied to blast against the database. Blastn with *e*-value cutoff 1,000 was performed to obtain short HSP hits with the complemented intron sequence. The result found 23 lncRNA hits, and those hits with the opposite orientation compared with the intron sequence (Plus/Minus) were filtered as the lncRNA, which could bind the transcriptional intron, but not directed on the DNA strand (Figure 7B).

The nervous system regulation network building from transcription factors that bind to the promotor region of *PTPRT* gene showed that *PTPRT* gene mainly participates in the positive regulation of programmed cell death and dephosphorylation, which are majorly involved in *SOD1*, *SQSTM1*, *VCP*, and *FIG4* genes; their related genes including *MATR3*, *HNRNPA1*, *TARDBP*, *ATXN2*, *UBQLN2*, *DCTN1*, *FUS*, *OPTN*, *VAPB*, *NEFH*, *PRPH*, *CHMP2B*, *ANG*, *TBK1*, and *PPARGC1A* genes; and KLF4, RELA, and MYOD1 transcription factors (Figure 8B).

### Gene Ontology Analysis of 10 Candidate Genes

Ten candidate genes were involved in 40 of the biological processes. Of them, DAB1-related biological processes included 36 of the biological processes, which mainly consisted of the negative regulation of cell adhesion, the phosphate-containing compound metabolic process (dephosphorylation), the cell development (neuron, cerebral cortex, forebrain, neuron projection, pallium, dendrite, and brain), the regulation of cell morphogenesis involved in differentiation, the regulation of neuron differentiation, the regulation of response to external stimulus, the regulation of neurogenesis, the locomotory and single-organism behavior, the differentiation, migration, generation recognition of neurons, neurogenesis, and the regulation of axonogenesis. PTPRT-related genes included six of the biological processes, which were the biological adhesion, the cell adhesion, the cell–cell adhesion, the phosphate-containing compound metabolic process, the phosphorus metabolic process, and dephosphorylation, which mainly participated in the adhesion and phosphorus metabolic processes (Supplementary Table 6 and Supplementary Figure 1). The commonly shared biological processes were the biological adhesion, the cell adhesion, the cell–cell adhesion, the phosphate-containing compound metabolic process, and the phosphorus metabolic process. It indicated that *DAB1* and *PTPRT* genes existed in very similar biological processes.

## Discussion

Previous studies suggest that genetic commonalities exist in the pathogenesis of sPD and sALS; however, these have yet to be identified. In this study, we found that *DAB1* rs17115303 and *PTPRT* rs6030462 mutations are common to sPD and sALS and may play causative roles in the pathogenesis of both diseases. This was confirmed by the results of the GO analysis, which showed that the two genes have overlapping functions in development, differentiation, neurogenesis, migration, axonogenesis, adhesion, and the metabolism of phosphate-containing compounds.

*DAB1* gene [also known as spinocerebellar ataxia type 37 (*SCA37*)] located on 1p32.2 consists of 21 exons and encodes the adaptor protein reelin. *DAB1* is expressed in many organs, with especially high expression in the small intestine, duodenum, and brain (Fagerberg et al., 2014). The laminar organization of the cerebral cortex is required for normal cognitive function; in the developing mouse brain, DAB1 protein directs the migration of newborn cortical neurons past previously formed layers to their final location *via* its role as a signal transducer in protein kinase pathways (Lee and D’Arcangelo, 2016). DAB1 binds to phosphatidylinositol 3-kinase and other proteins (Rolland et al., 2014) and has been implicated in many biological processes including adult walking behavior (Strazielle et al., 2012); axon guidance (Yamashita et al., 2006; Palmesino et al., 2010); radial glia-guided neuronal migration in the cerebral cortex (Franco et al., 2011); organization of cerebellum structure (Gallagher et al., 1998); dendrite development (Niu et al., 2004); migration of lateral motor column neurons (Palmesino et al., 2010); negative regulation of astrocyte differentiation (Kwon et al., 2009), axonogenesis (Matsuki et al., 2008), and cell adhesion (Koch et al., 2002; Feng et al., 2007); positive regulation of neuron differentiation (Tabata and Nakajima, 2002) and protein kinase activity (Lee and D’Arcangelo, 2016); radial glia-guided migration of Purkinje cells; and small GTPase-mediated signal transduction and ventral spinal cord development (Goffinet et al., 1999; Vaswani and Blaess, 2016). *DAB1* rs17115303 in this study was found to be related to the pathogenesis of sPD and sALS and was their common risk factor; this mutation might change some above-described functions of *DAB1* and participate in the development and progression of sPD and sALS.

*PTPRT* gene (also known as *RPTPrho*) located on 20q12–q13.11 consists of 37 exons and is mainly expressed in the brain and placenta (Fagerberg et al., 2014). The protein encoded by this gene is a protein tyrosine phosphatase that is thought to be involved in signal transduction and cell adhesion in the central nervous system. Two splice variants of this gene have been reported. *PTPRT* is known to be involved in binding to α-, β-, γ-, and δ-catenin and cadherin, cell adhesion, protein dephosphorylation, signal transduction, and transmembrane receptor protein tyrosine kinase signaling (Besco et al., 2006); protein binding, protein phosphatase binding, protein homodimerization activity, transmembrane receptor protein tyrosine phosphatase activity, negative regulation of cell migration, and peptidyl-tyrosine dephosphorylation in the inactivation of protein kinase activity (Qi et al., 2014); signal transducer and activator of transcription (STAT) protein binding, the cellular response to interleukin-6 (IL-6), and peptidyl-tyrosine dephosphorylation (Zhang et al., 2007); protein tyrosine phosphatase activity (Besco et al., 2006; Zhang et al., 2007); negative regulation of the STAT cascade (Besco et al., 2006; Zhang et al., 2007; Qi et al., 2014); and homophilic cell adhesion *via* plasma membrane adhesion molecules (Yu et al., 2008). *PTPRT* plays some important physiological effects in protein metabolism; *PTPRT* rs6030462 mutations might affect some important protein phosphate, transmembrane transportation, and dephosphorylation to result in the degradation disorder of some neuron-proteins, subsequently producing some toxic proteins such as α-synuclein, TDP43, and FUS/TLS, which damage neural cells, contributing to sPD and sALS.

The MAF of *DAB1* rs17115303 was significantly higher in both sPD and sALS patients than in control subjects. The minor A allele of rs17115303 was associated with an increased risk for both sPD and sALS, while the C allele was associated with a decreased risk. Additionally, carriers of the A allele of *PTPRT* rs6030462 had a higher risk of both sPD and sALS, whereas those of the G allele had a decreased risk. We suggested that *DAB1* rs17115303 and *PTPRT* rs6030462 polymorphisms are genetic risk factors common to both diseases that directly or indirectly promote the accumulation of intraneuronal inclusions or neurodegeneration of DA and motor neurons, resulting in movement disorder. Recent evidence also suggests dysregulation of serotonergic neurotransmission (Vermeiren et al., 2018) and lymphoproliferative disorder (Puri and Carrithers, 2019) as possible mechanistic links between PD and ALS. Additional studies are needed to clarify the detailed roles of *DAB1* or *PTPRT* in the etiology of these two diseases and to determine whether the same therapeutic strategies can be applied to their treatment. The common variations in the genes of mitochondria function contribute to the heritable component of sPD; the specific polygenic risk scores of mitochondria functions are shown to significantly associate with sPD status and are significantly related to the later age of sPD onset. The Mendelian random study finds 14 novel mitochondria function genes of possible pathogenic relationship with sPD risk (Billingsley et al., 2019). Because the common genetic risks exist in both sPD and sALS, research about the mitochondria function genes associated with the pathogenesis of both sPD and sALS will have a very promising way for exploring the common pathogenesis of both sPD and sALS.

## Conclusion

Variants of *DAB1* gene (rs17115303) and *PTPRT* gene (rs6030462) are common risk factors to sPD and sALS in the HPMC. These two genes are involved in the functions of development, differentiation, neurogenesis, migration, axonogenesis, adhesion, and the metabolism of phosphate-containing compounds. This study suggests that the pathogenesis of sPD and sALS exists through multiple common potential similar factors.

## Limitation

While there was poor replication of GWAS findings in PD and ALS between Caucasians and Chinese (Yang et al., 2019; Iacoangeli et al., 2020), which indicated the different genetic architectures in the two populations, it may not be an optimal design to combine Caucasian risk loci of PD with Chinese risk loci of ALS to explore the potential common risk loci. If the Asian or Chinese Han populations for the investigated subject are selected, this study would be more rational. Additionally, this study has a small sample and lacks replicated samples.

## Data Availability Statement

The original contributions presented in the study are included in the article/Supplementary Material, further inquiries can be directed to the corresponding author.

## Ethics Statement

The studies involving human participants were reviewed and approved by the institutional review board of hospital human Ethics Committee of First Affiliated Hospital of Nanchang University. All experimental methods complied with Helsinki Declaration. The patients/participants provided their written informed consent to participate in this study.

## Author Contributions

YL, WC, CW, and RX designed the studies and wrote and edited the manuscript. YL, WC, CW, and YZ carried out the experiments and analyzed the data. All authors contributed to the article and approved the submitted version.

## Conflict of Interest

The authors declare that the research was conducted in the absence of any commercial or financial relationships that could be construed as a potential conflict of interest.

## Publisher’s Note

All claims expressed in this article are solely those of the authors and do not necessarily represent those of their affiliated organizations, or those of the publisher, the editors and the reviewers. Any product that may be evaluated in this article, or claim that may be made by its manufacturer, is not guaranteed or endorsed by the publisher.

## Footnotes

1
http://www.ncbi.nlm.nih.gov/gap/

2
https://useast.ensembl.org/Homo_sapiens/Info/Index

3
www.omim.org

4
http://geneontology.org/

## Supplementary Material

Supplementary Figure 1 GO analysis of ten candidate genes. Ten candidate genes involved in 40 significantly correlated biological processes. *DAB1* was involved in 36 biological processes and *PTPRT* was involved in six. Related biological processes are shown in black square boxes in red typeface.
