# Supplementary figures and images for: Potential Common Genetic Risks of Sporadic Parkinson’s Disease and Amyotrophic Lateral Sclerosis in the Han Population of Mainland China
Source: Front Neurosci. 2021 Oct 11;15:753870. doi: 10.3389/fnins.2021.753870 (PMC8542930; doi:10.3389/fnins.2021.753870)

## Supplementary figure 1

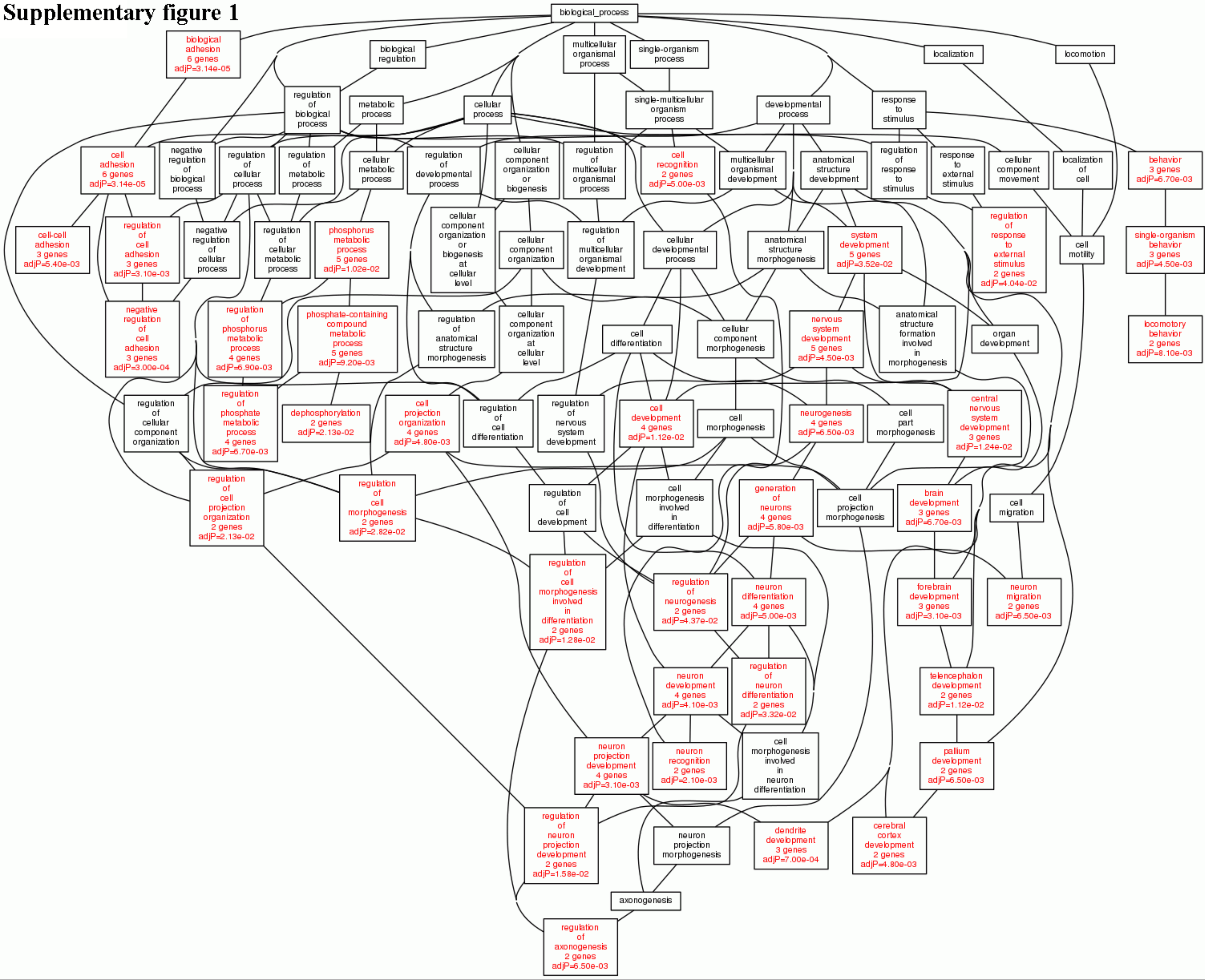

Supplement: Supplementary Figure 1 — GO analysis of ten candidate genes. Ten candidate genes involved in 40 significantly correlated biological processes. DAB1 was involved in 36 biological processes and PTPRT was involved in six. Related biological processes are shown in black square boxes in red typeface. [file Image_1.PDF]
