# Supplementary material for: Potential Common Genetic Risks of Sporadic Parkinson’s Disease and Amyotrophic Lateral Sclerosis in the Han Population of Mainland China
Source: Front Neurosci. 2021 Oct 11;15:753870. doi: 10.3389/fnins.2021.753870 (PMC8542930; doi:10.3389/fnins.2021.753870)
Supplement: Supplementary file 2 [file Table_1.DOC]

**Supplementary Table 1 The basic information of PD study from dbGAP**

| Study | Sample size | Genes | |
| --- | --- | --- | --- |
|  | (Case/Control) | SNPs | *P*< 10^-2^ |
| Study 1 ^[1]^ | 816/856 | 43,474 | 2,604 |
| Study 2 ^[2]^ | Stage1：1713/3978  Stage2：3452/4756 | 44,304 | 5,693 |
| Study 3 ^[3]^ | 269/266 | 43,699 | 3,782 |
| Study 4 ^[4]^ | Tier1：443/443  Tier2：332/332 | 41,458 | 1,407 |
| Over-lap | - | 2,779 | 52 |

References: [1] Pankratz N, Dumitriu A, Hetrick KN, et al., Copy number variation in familial Parkinson disease. PLoS One. 2011; 6(8): e20988. [2] [Simón-Sánchez J, Schulte C, Bras JM,](http://www.ncbi.nlm.nih.gov/sites/entrez?db=pubmed&cmd=DetailsSearch&term=19915575%5BPMID%5D) et al., Genome-wide association study reveals genetic risk underlying Parkinson's disease. Nat Genet. 2009; 41(12): 1308-12. [3] [Fung HC, Scholz S, Matarin M, et al.,](http://www.ncbi.nlm.nih.gov/sites/entrez?db=pubmed&cmd=DetailsSearch&term=17052657%5BPMID%5D) Genome-wide genotyping in Parkinson's disease and neurologically normal controls: first stage analysis and public release of data. Lancet Neurol. 2006; 5(11): 911-6. [4] [Maraganore DM, de Andrade M, Lesnick TG, et al.,](http://www.ncbi.nlm.nih.gov/sites/entrez?db=pubmed&cmd=DetailsSearch&term=16252231%5BPMID%5D) High-resolution whole-genome association study of Parkinson disease. Am J Hum Genet. 2005; 77(5): 685-93.
