# Supplementary material for: Potential Common Genetic Risks of Sporadic Parkinson’s Disease and Amyotrophic Lateral Sclerosis in the Han Population of Mainland China
Source: Front Neurosci. 2021 Oct 11;15:753870. doi: 10.3389/fnins.2021.753870 (PMC8542930; doi:10.3389/fnins.2021.753870)
Supplement: Supplementary file 3 [file Table_2.DOC]

**Supplementary Table 2** The P-value of ten common genes identified from dbGAP datasets and ALS patients

| *P*-value | | | | | | | | | |
| --- | --- | --- | --- | --- | --- | --- | --- | --- | --- |
| Gene | *Study1* | | *Study 2* | | | *Study 3* | | *Study 4* | *ALS* |
|  | pha002865 | pha002876 | pha003127 | pha003128 | pha000003 | pha000004 | pha002868 | pha002840 | ALS |
| CNTNAP2 | 0.0003491 | 0.002429 | 0.001475263 | 0.002454588 | 0.0006411 | 0.002635155 | 0.001212 | 0.008151 | 3.68E-05 |
| CSMD1 | 0.0008205 | 0.0005878 | 0.000124419 | 0.001061413 | 0.004217 | 0.001618255 | 0.000001852 | 0.004231 | 2.42E-07 |
| DAB1 | 0.00557 | 0.001135 | 0.000415955 | 0.00464408 | 0.001617 | 0.002431876 | 0.003092 | 0.0022 | 9.27E-04 |
| DSCAM | 0.0006618 | 0.0008101 | 0.002743352 | 0.005546445 | 0.004146 | 0.008363346 | 0.001599 | 0.005837 | 5.70E-04 |
| LSAMP | 0.003866 | 0.001119 | 0.00077776 | 2.17E-05 | 0.003889 | 0.00103358 | 0.000892 | 0.007231 | 2.71E-03 |
| PRKG1 | 0.009788 | 0.002648 | 0.000463066 | 0.0075956 | 0.002425 | 0.005418483 | 0.0006003 | 0.005159 | 4.04E-04 |
| PTPRT | 0.003481 | 0.008248 | 0.006313225 | 0.001804342 | 0.0004294 | 0.000324695 | 0.003664 | 0.006741 | 1.95E-04 |
| STK32B | 0.001504 | 0.002855 | 0.007911464 | 0.00574855 | 0.005547 | 0.003043872 | 0.003951 | 0.0007005 | 6.88E-04 |
| TMEM132D | 0.0008601 | 0.006881 | 0.000721246 | 0.001080107 | 0.001743 | 0.002150316 | 0.0005514 | 0.003027 | 1.48E-04 |
| ZMAT4 | 0.004617 | 0.007589 | 0.002632193 | 0.003371021 | 0.001669 | 0.005365602 | 0.0008157 | 0.002253 | 1.02E-05 |
