# Supplementary material for: Potential Common Genetic Risks of Sporadic Parkinson’s Disease and Amyotrophic Lateral Sclerosis in the Han Population of Mainland China
Source: Front Neurosci. 2021 Oct 11;15:753870. doi: 10.3389/fnins.2021.753870 (PMC8542930; doi:10.3389/fnins.2021.753870)
Supplement: Supplementary file 4 [file Table_3.DOC]

**Supplementary Table 3** Genes from reported OMIM studies

| Gene | OMIM ID |
| --- | --- |
| CNTNAP2 | 604569 |
| CSMD1 | 608397 |
| DAB1 | 603448 |
| DSCAM | 602523 |
| LSAMP | 603241 |
| PRKG1 | 176894 |
| PTPRT | 608712 |
| STK32B | \ |
| TMEM132D | 611257 |
| ZMAT4 | \ |
