# Supplementary material for: Potential Common Genetic Risks of Sporadic Parkinson’s Disease and Amyotrophic Lateral Sclerosis in the Han Population of Mainland China
Source: Front Neurosci. 2021 Oct 11;15:753870. doi: 10.3389/fnins.2021.753870 (PMC8542930; doi:10.3389/fnins.2021.753870)
Supplement: Supplementary file 5 [file Table_4.DOC]

**Supplementary Table 4** All SNPs were chosen in the association study

| Gene | Chr | SNPs | Position |
| --- | --- | --- | --- |
| CNTNAP2 | 7 | rs17434745 | 147989522 |
| CSMD1 | 8 | kgp12078483(rs62484656) | 4754792 |
| DAB1 | 1 | kgp15539347(rs17115303) | 57194802 |
| DSCAM | 21 | rs2837371 | 40011216;41383142 |
|  | 21 | rs8130587 | 41865052 |
|  | 21 | rs2837740 | 41967515 |
| LSAMP | 3 | rs9874470 | 116246801;115965647 |
|  | 3 | kgp1203481(rs73139176) | 115758478 |
|  | 3 | rs868114 | 116900418 |
| PRKG1 | 10 | rs17560636 | 53803755 |
|  | 10 | rs16916942 | 53100259 |
| PTPRT | 20 | rs1884040 | 42077854;40706493 |
|  | 20 | kgp10150212 (rs4812690) | 41816349 |
|  | 20 | rs6030462 | 41400229 |
| STK32B | 4 | kgp10356021(rs111466658) | 5186428 |
|  | 4 | rs195112 | 5413698 |
| TMEM13D | 12 | rs2218917 | 130224189 |
|  | 12 | rs4609642 | 130229897 |
|  | 12 | rs11060391 | 130007355 |
|  | 21 | rs2837170 | 41130447 |
| ZMAT4 | 8 | rs11782411 | 40336076 |
|  | 8 | rs2589916 | 40382480 |

Abbreviations: Chro=Chromosome; SNPs=Single nucleotide polymorphisms.
