# Supplementary material for: Potential Common Genetic Risks of Sporadic Parkinson’s Disease and Amyotrophic Lateral Sclerosis in the Han Population of Mainland China
Source: Front Neurosci. 2021 Oct 11;15:753870. doi: 10.3389/fnins.2021.753870 (PMC8542930; doi:10.3389/fnins.2021.753870)
Supplement: Supplementary file 6 [file Table_5.DOC]

**Supplementary Table 5** Demographic characteristics of sPD participates

| Demographic characteristics | Case  (n=530) | Control  (n=530) |
| --- | --- | --- |
| Average age at onset (cases) or at enrollment (controls) | 58.72±0.91 | 59.56±0.92 |
| Median age (range) at onset | 60(32-83) | 60(31-83) |
| Age <40 | 18 | 16 |
| Age <50 | 94 | 84 |
| Age <60 | 166 | 156 |
| Age <70 | 155 | 159 |
| Age <80 | 90 | 106 |
| Age ≥80 | 7 | 9 |
| % males | 51.70% | 60% |
