# Supplementary material for: Potential Common Genetic Risks of Sporadic Parkinson’s Disease and Amyotrophic Lateral Sclerosis in the Han Population of Mainland China
Source: Front Neurosci. 2021 Oct 11;15:753870. doi: 10.3389/fnins.2021.753870 (PMC8542930; doi:10.3389/fnins.2021.753870)
Supplement: Supplementary file 7 [file Table_6.DOC]

**Supplementary Table 6** The gene ontology analysis of ten genes

| Title of processes | NRG(GO) | NGG | | EN | RE | R | rawP | adjP | Title of related genes |
| --- | --- | --- | --- | --- | --- | --- | --- | --- | --- |
| biological adhesion | 0022610 | 956 | | 6 | 0.46 | 13.12 | 5.07e-07 | 3.14e-05 | DAB1,CNTNAP2,DSCAM,LSAMP,PTPRT,PRKG1 |
| cell adhesion | 0007155 | 954 | | 6 | 0.46 | 13.14 | 5.01e-07 | 3.14e-05 | DAB1,CNTNAP2,DSCAM,,LSAMP,PTPRT,PRKG1 |
| negative regulation of cell adhesion | 0007162 | 86 | | 3 | 0.04 | 72.91 | 6.75e-06 | 0.0003 | DAB1,DSCAM,PRKG1 |
| dendrite development | 0016358 | 126 | | 3 | 0.06 | 49.76 | 2.13e-05 | 0.0007 | DAB1,DSCAM,PRKG1 |
| neuron recognition | 0008038 | 30 | | 2 | 0.01 | 139.33 | 8.48e-05 | 0.0021 | CNTNAP2,DSCAM |
| regulation of cell adhesion | 0030155 | 264 | | 3 | 0.13 | 23.75 | 0.0002 | 0.0031 | DAB1,DSCAM,PRKG1 |
| neuron projection development | 0031175 | 704 | | 4 | 0.34 | 11.88 | 0.0002 | 0.0031 | DAB1,CNTNAP2,DSCAM,PRKG1 |
| forebrain development | 0030900 | 281 | | 3 | 0.13 | 22.31 | 0.0002 | 0.0031 | DAB1,CNTNAP2,PRKG1 |
| neuron development | 0048666 | 798 | | 4 | 0.38 | 10.48 | 0.0003 | 0.0041 | DAB1,CNTNAP2,DSCAM,PRKG1 |
| single-organism behavior | 0044708 | 334 | | 3 | 0.16 | 18.77 | 0.0004 | 0.0045 | DAB1,CNTNAP2,DSCAM |
| nervous system development | 0007399 | 1724 | | 5 | 0.82 | 6.06 | 0.0004 | 0.0045 | DAB1,CNTNAP2,DSCAM,LSAMP,PRKG1 |
| cerebral cortex development | 0021987 | 72 | | 2 | 0.03 | 58.06 | 0.0005 | 0.0048 | DAB1,CNTNAP2 |
| cell projection organization | 0030030 | 934 | | 4 | 0.45 | 8.95 | 0.0005 | 0.0048 | DAB1,CNTNAP2,DSCAM,PRKG1 |
| cell recognition | 0008037 | 77 | | 2 | 0.04 | 54.29 | 0.0006 | 0.0050 | CNTNAP2,DSCAM |
| neuron differentiation | 0030182 | 990 | | 4 | 0.47 | 8.44 | 0.0006 | 0.0050 | DAB1,CNTNAP2,DSCAM,PRKG1 |
| cell-cell adhesion | 0016337 | 404 | | 3 | 0.19 | 15.52 | 0.0007 | 0.0054 | DAB1,PTPRT,PRKG1 |
| generation of neurons | 0048699 | 1073 | | 4 | 0.51 | 7.79 | 0.0008 | 0.0058 | DAB1,CNTNAP2,DSCAM,PRKG1 |
| neurogenesis | 0022008 | 1140 | | 4 | 0.55 | 7.33 | 0.0011 | 0.0065 | DAB1,CNTNAP2,DSCAM,PRKG1 |
| pallium development | 0021543 | 106 | | 2 | 0.05 | 39.43 | 0.0011 | 0.0065 | DAB1,CNTNAP2 |
| regulation of axonogenesis | 0050770 | 103 | | 2 | 0.05 | 40.58 | 0.0010 | 0.0065 | DAB1,DSCAM |
| neuron migration | 0001764 | 101 | | 2 | 0.05 | 41.39 | 0.0010 | 0.0065 | DAB1,PRKG1 |
| brain development | 0007420 | 502 | | 3 | 0.24 | 12.49 | 0.0013 | 0.0067 | DAB1,CNTNAP2,PRKG1 |
| behavior | 0007610 | 508 | | 3 | 0.24 | 12.34 | 0.0013 | 0.0067 | DAB1,CNTNAP2,DSCAM |
| regulation of phosphate metabolic process | 0019220 | 1204 | | 4 | 0.58 | 6.94 | 0.0013 | 0.0067 | TMEM132D,DAB1,DSCAM,PRKG1 |
| regulation of phosphorus metabolic process | 0051174 | 1216 | | 4 | 0.58 | 6.88 | 0.0014 | 0.0069 | TMEM132D,DAB1,DSCAM,PRKG1 |
| locomotory behavior | 0007626 | 136 | | 2 | 0.07 | 30.74 | 0.0017 | 0.0081 | DAB1,DSCAM |
| phosphate-containing compound metabolic process | 0006796 | 2438 | | 5 | 1.17 | 4.29 | 0.0020 | 0.0092 | TMEM132D,DAB1,DSCAM,PTPRT,PRKG1 |
| phosphorus metabolic process | 0006793 | 2501 | | 5 | 1.20 | 4.18 | 0.0023 | 0.0102 | TMEM132D,DAB1,DSCAM,PTPRT,PRKG1 |
| cell development | 0048468 | 1461 | | 4 | 0.70 | 5.72 | 0.0027 | 0.0112 | DAB1,CNTNAP2,DSCAM,PRKG1 |
|  | | | | | | | | | |
|  |  |  |  | |  |  |  |  |  |
| Cont. |  |  |  | |  |  |  |  |  |
| telencephalon development | 0021537 | 168 | 2 | | 0.08 | 24.88 | 0.0027 | 0.0112 | DAB1,CNTNAP2 |
| central nervous system development | 0007417 | 688 | 3 | | 0.33 | 9.11 | 0.0031 | 0.0124 | DAB1,CNTNAP2,PRKG1 |
| regulation of cell morphogenesis involved in differentiation | 0010769 | 187 | 2 | | 0.09 | 22.35 | 0.0033 | 0.0128 | DAB1,DSCAM |
| regulation of neuron projection development | 0010975 | 213 | 2 | | 0.10 | 19.62 | 0.0042 | 0.0158 | DAB1,DSCAM |
| dephosphorylation | 0016311 | 252 | 2 | | 0.12 | 16.59 | 0.0059 | 0.0213 | TMEM132D,PTPRT |
| regulation of cell projection organization | 0031344 | 256 | 2 | | 0.12 | 16.33 | 0.0060 | 0.0213 | DAB1,DSCAM |
| regulation of cell morphogenesis | 0022604 | 300 | 2 | | 0.14 | 13.93 | 0.0082 | 0.0282 | DAB1,DSCAM |
| regulation of neuron differentiation | 0045664 | 331 | 2 | | 0.16 | 12.63 | 0.0099 | 0.0332 | DAB1,DSCAM |
| system development | 0048731 | 3521 | 5 | | 1.68 | 2.97 | 0.0108 | 0.0352 | DAB1,CNTNAP2,DSCAM,LSAMP,PRKG1 |
| regulation of response to external stimulus | 0032101 | 376 | 2 | | 0.18 | 11.12 | 0.0127 | 0.0404 | DSCAM,PRKG1 |
| regulation of neurogenesis | 0050767 | 397 | 2 | | 0.19 | 10.53 | 0.0141 | 0.0437 | DAB1,DSCAM |

**Comment:** NRG: number of reference genes in category. NGG: number of genes in gene set and also in category. EN: expected number in category. RE: ratio of enrichment, rawP: p value from hypergeometric test, adjP: p value adjusted by multiple test adjustment.
